# Evaluation of tomato based agro-industrial byproducts as substrates for *Trichoderma harzianum* cultivation and bioinoculant potential

**DOI:** 10.3389/fmicb.2025.1713960

**Published:** 2026-01-16

**Authors:** Antonia Esposito, Valeria Scala, Nikolay Vassilev, Maria Aragona, Loredana Canfora, Alessandro Polito, Riccardo Fiorani, Stefano Mocali

**Affiliations:** 1 Research Centre for Agriculture and Environment, Council for Agricultural Research and Economics (CREA-AA), Florence, Italy; 2 Research Centre for Plant Protection and Certification, Council for Agricultural Research and Economics (CREA-DC), Rome, Italy; 3 Department of Chemical Engineering, Institute of Biotechnology, University of Granada, Granada, Spain; 4 Research Centre for Agriculture and Environment, Council for Agricultural Research and Economics (CREA-AA), Rome, Italy

**Keywords:** agro-industrial waste, biocontrol agent, biomass yield, gazpacho, multifunctional bioinoculant, salt stress tolerance, sustainable production, *Trichoderma harzianum*

## Abstract

*Trichoderma harzianum* is a well-known biocontrol agent with growing interest as a multifunctional bioinoculant due to its diverse metabolic capabilities. Despite its promising potential, the transition from laboratory-scale cultivation to industrial-scale production still presents challenges, particularly in optimizing biomass and spore yield at low cost. This study focused on testing a new medium for spore/mycelium production of *T. harzianum* integrating traditional growth media with gazpacho, a tomato-based by-product of Andalusian food as cheap substrate. We also assessed its multifunctional activity, including the tolerance to salt stress, solubilization of rock phosphate and the antagonistic activity against three major tomato pathogens (*Botrytis cinerea*, *Fusarium oxysporum*, and *Pyrenochaeta lycopersici*) through dual culture assays. The results showed that media supplemented with 3 and 6% (v/v) gazpacho significantly increased *T. harzianum* biomass and sporulation in solid and submerged state fermentations, while 10% reduced spore formation in liquid submerged fermentation. Interestingly, biomass and sporulation were further improved in media containing 3–6% (v/v) gazpacho combined with 100 mM NaCl. *Trichoderma harzianum* was able to grow and sporulate in solid media with up to 100 mM NaCl. Moreover, the strain showed phosphate solubilization activity on gazpacho-containing media in submerged fermentation, and effectively inhibited over 70% of pathogenic mycelial growth, with *B. cinerea* showing the highest inhibition (78.40%). Overall, these results highlight the improvement in biomass and spore production of *T. harzianum* grown in traditional growth media supplemented with 6% gazpacho, as well as its multifunctional activities under these fermentation conditions, thus representing a promising approach towards the production of cheap bioinoculants and supporting the circular economy in microbial technology. Furthermore, salt tolerance further encourage *T. harzianum* as a robust candidate for bioformulations in challenging agro-environment.

## 1 Introduction

Fertilizers are substances that improve the nutrients in the soil, enabling plants to grow. In the last few decades, fertilization and pest control in agriculture have been mainly achieved using synthetic chemical products, meeting the high demand for production to sustain increasing populations. However, their use has caused bioaccumulation and persistence in the soil, the loss of rhizosphere biological activity in terms of microbiome richness and diversity, the emergence of resistant pathogens to fungicides, leading to severe adverse effects on food safety and human health (Zhu et al., 2018). Hence, research has redirected to study alternative approaches that minimize negative impacts on both human health and the environment and, at least partially, replace mineral fertilizers (Latini et al., 2021).

Plant beneficial microorganisms were shown to be a safer alternative to mineral fertilizers and as a part of sustainable agriculture and scientific efforts to promote healthier soil and food (Vassileva et al., 2021). Microorganisms with industrial and commercial interest, which demonstrate plant beneficial properties, are mainly bacteria (*Bacillus*, *Pseudomonas*, *Rhizobium*, and *Azotobacter*, among others) and fungi (*Aspergillus*, *Penicillium, Trichoderma*, *Beauveria*, *Metarhizium*, *Clonostachys*, and mycorrhizal fungi). These microorganisms are well known for enhancing plant growth through numerous mechanisms, including the suppression of plant pathogens (Whipps, 1997), enhancing the availability of nutrients to the host plant, solubilizing insoluble nutrients and enhancing the production of stimulatory compounds, such as plant growth regulators (Whipps, 1997; Antoun and Prévost, 2005). It should be noted that many of these microorganisms manifest more than one of the above-mentioned activities, as they possess multifunctional properties. An example is represented by *Trichoderma* species, including filamentous fungi commonly found in soil worldwide (Vinale et al., 2008; Schmoll and Schuster, 2010). Among these, *Trichoderma harzianum* is well known as a biocontrol agent against a wide range of plant pathogens, being used for developing promising biological fungicides in agriculture. In addition to protecting crops, it is widely recognized for its beneficial effects on crops by increasing the availability of nutrients, improvement of plant resilience against abiotic stresses, such as salinity and drought, and facilitation of beneficial microbial interactions in the rhizosphere, underlining an effective plant growth promoting fungi (PGPF) (Kumar A. et al., 2023).

Nevertheless, in the industrial production of fungal bioinoculants, processing steps such as the production of high biomass and spores, formulation, and storage, are all limiting points able to reduce the number and the vitality of the microorganisms (Vassileva et al., 2021). Moreover, cost-effectiveness may hinder promising microorganisms from becoming products. Notably, it has been reported that the main process variables affecting the economic feasibility of large-scale production of biofertilizers are related to cultivation steps due to the substrate and biofertilizer composition (Vassileva et al., 2021). In fact, the cost of synthetic media per liter varied from 1.2 to 3.2 $ (Chand Kumhar et al., 2014; Machado et al., 2018). Thus, considering that the volumes usually adopted for industrial-scale production may vary up to 10-30 m^3^ (Kumar V. et al., 2023), the high cost of culture media leads to propose some strategies to overcome this drawback aiming its industrial production. Consequently, interest has grown in residues and/or byproducts from agroindustry and food industry as carbon and nitrogen sources for new compositions of culture media (Tengerdy and Szakács, 1998; Vassileva et al., 2010). Several authors have reported that the use of “low-cost” components has reduced the cost of culture media by 62–86% and biomass production was similar or higher than that of commercial media (Bapat et al., 2003; Valle Vargas et al., 2024).

Several studies on optimizing biomass and spore production using cheap substrates or agro-industrial waste have been reported, especially for industrial inoculant production (Vassileva N. et al., 2009; Vassileva et al., 2024; Sala et al., 2019; Vassileva et al., 2021). For example, sugar beet (SB), cheese whey, malt sprouts, and molasses were included in media composition and showed high suitability in large-scale production conditions with additional cell-protective advantages demonstrated in the formulation stage (Vassileva et al., 2010; Vassileva et al., 2021). However, at the best of our knowledge, tomato by-products have never been evaluated as alternative growing media for microbial production (Almeida et al., 2024). Nevertheless, among fruits and vegetables, tomato is one of the most widespread crops in the world, with a global annual production that exceeded 180 million tons in 2021 (Eslami et al., 2023). Italy and Spain, with a production that, in 2023, reached about 10 million tons, account for 75% of the total European production (Agricultural Production - Crops - Statistics Explained - Eurostat, 2024). During tomato processing, huge quantities of by-products are generated, accounting for 3–5% (w/w) of the total raw tomatoes. Therefore, in view of a large-scale, cost-effective production of eco-friendly bioinoculum, the present preliminary work was carried out to evaluate tomato by-products as supplementary source for standard culture media for the mass multiplication of *T. harzianum*, and to explore its multifunctional potential for application in tomato cultivation.

In order to achieve such goals, we first assessed the influence of varying tomato by-products concentrations on the *T. harzianum* growth under both solid-state and liquid submerged fermentation conditions. Secondly, we evaluated some of multiple beneficial activities *in vitro* of *T. harzianum* including: (i) its antagonistic activity against three phytopathogenic fungi for tomato, (ii) its capacity to solubilize rock phosphate (RP), and (iii) its tolerance to different salt stress on media with and without gazpacho supplementation.

## 2 Materials and methods

### 2.1 Fungal strains

The *Trichoderma harzianum* strain (available at the Spanish Type Culture Collection, identifier: CECT 20727, designation NBT-85) used in this study was provided by the Department of Chemical Engineering at the University of Granada and was maintained on potato dextrose agar (PDA) at 4 °C. Three tomato fungal pathogens were used for testing antagonistic activity of *T. harzianum*: *Botrytis cinerea* ER 1235, *Fusarium oxysporum forma specialis lycopersici and Pyrenochaeta lycopersici* ER 1211. The fungal strains were from the collection of CREA-DC in Rome, they were maintained on culture tubes filled with PDA under vaselin oil at 4 °C.

### 2.2 Culture media

Considering the variability in the composition of tomato by-products, we used a commercial gazpacho (Hacendado, JGC S. A., Madrid, Spain), —a liquid mixture primarily composed of tomatoes and other vegetable residues—as the tomato by-product source. Gazpacho (lot: LH2845E purchase date: 18.09.24) was bought from a local supermarket and immediately used. The remaining gazpacho was kept at 4 °C until subsequent use. The gazpacho has the following composition (g·L^−1^): 24 g lipids, 36 g carbohydrates of which 34 g sugars, 11 g fiber, 8 g proteins, 8 g salt, 4.2 pH. Potato dextrose broth (PDB) and potato dextrose agar (PDA) used contain per liter of distilled water (g·L^−1^): 4 g potato extract, 20 g dextrose, and 15 g agar for PDA only. For both media 3, 6 and 10% (v/v) gazpacho was added. Czapeck-dox modified agar used contains per liter of distilled water: 30 g sucrose, 2 g sodium Nitrate, 0.5 g magnesium glycerophosphate, 0.5 g potassium chloride, 0.35 g potassium sulfate, 0.01 g ferrous sulfate, 12 g bacteriological agar. All media were autoclaved at 121 °C for 20 min.

### 2.3 Experimental conditions

Experiments in condition of solid-state fermentation (SSF) were performed on PDA. A block of 5 mm of *Trichoderma* mycelium of a 7-day-old culture was used for inoculation on PDA. The block was cut with a sterilized cork borer from the edge of the culture. Each mycelial block was placed upside down at the centre of each plate. Incubation of the fungal culture was carried out at 28 ± 2 °C for 7 days. Three replicates were used for each medium. The fermentation in the condition of submerged liquid process (SmF) was carried out in 250 mL Erlenmeyer flasks containing 100 mL of fermentation medium (PDB) inoculated with 1 mL of fungal inoculum (10^8^ spores mL^−1^), obtaining a final concentration of 10^6^ spores mL^−1^. Fungal inoculum was prepared by harvesting spore from 7-day-old *T. harzianum* cultures grown on PDA supplemented with 0.1% (v/v) Tween 80 solution. The inoculated conical flasks were kept in the shaker incubator at 28 ± 2 °C for 7 days. The growth of *T. harzianum*, in term of mycelium biomass and spore production, was performed in SSF and SmF by assessing: (i) the effect of different concentrations of gazpacho (3, 6, 10% [v/v]) (ii) the effect of 100 mM of NaCl on medium containing gazpacho in submerged fermentation (iii) the solubilization of rock phosphate (RP) by *T. harzianum* in presence of gazpacho in submerged fermentation conditions.

### 2.4 Effect of NaCl on *T. harzianum* grown on solid media

The effect of NaCl was evaluated by exposing *T. harzianum* to gradients of sodium chloride (50 mM, 100 mM, 150 mM) on PDA medium in petri dishes. Negative control was only PDA medium. Tree replicates for each condition were used. The growth diameter was recorded 7 days after inoculation.

### 2.5 Average linear growth rate (ALGR) of *T. harzianum* and colony characteristics under solid-state fermentation (SSF) at different concentrations of gazpacho

Linear growth (mm) of *T. harzianum* was recorded every day until the mycelium covered the Petri dishes. Linear growth measured by averaging two diameters taken from each colony. Average linear growth rate was measured using the following formula: ALGR (mm/day) = (C1-C0)/3. Where C1 = Colony diameter after 3 days of inoculation C0 = Initial colony diameter (Aneja, 1993; Elad et al., 1981). Different colony characters such as surface, color, margin, texture and hyphal thickness were observed visually in each different media 7 days after incubation.

### 2.6 Evaluation of effect of gazpacho and NaCl on *T. harzianum* growth under submerged fermentation condition (SmF)

The effect of gazpacho and 100 mM NaCl on *T. harzianum* growth under SmF was evaluated at different days, by measuring the mean dry biomass and spore production. A volume of 1 mL of spore suspension was collected from each media at 5 and 7 days from inoculation and spores were counted by Thoma hemocytometer. After 7 days, mycelium was separated from liquid culture by a pre–weighted Whatman paper filter and air dried at 70 °C to obtain dry mass. The culture filtrates were used for pH measurement. Then the dried fungal biomass was weighed (g·L^−1^) by electronic balance.

### 2.7 Dual culture assay *in vitro*

A dual culture technique was used to evaluate the antagonistic efficacy of *T. harzianum* against different species of tomato pathogenic fungi (*B. cinerea*, *F. oxysporum* and *P. lycopersici*). Four-millimeter mycelial disks of the fungal pathogens and antagonistic strains were cut from the margin of the plates and were placed onto Czapeck-dox agar plates concurrently on opposite side (2,5 cm from the margin). The fungal pathogens were also cultured onto Czapeck-dox agar plates as controls and incubated at 25 ± 2 °C for 5 days. For each combination, five plates were used and incubated at 25 °C. Interactions were examined daily. The radial growth of the pathogenic strains on both dual cultures was measured using calipers. When mycelium of fungal pathogens and of *T. harzianum* in dual culture plate came into contact, the growth inhibition percentages were calculated, according to the following equation:

$$\%\text{Inhibition}=(\frac{A-B}{A})*100$$

Where A is the diameter of the phytopathogen colonies on control plates and B is the diameter of phytopathogen colonies on dual-culture plates. The results were expressed as a mean of five plates ± standard error.

### 2.8 Tomato seedlings-soil experiment in pot scale condition

Preliminary seedlings assay consisted of a pots experiment with tomato plants (*Solanum Lycopersicum L*., cv. Cuore di Bue) carried out in a greenhouse under natural conditions, without controlled temperature, light, or humidity (Esposito et al., 2025). Seedlings of tomato plants of uniform size (15 cm in height, 10 leaves) were transplanted into 4-L pots filled with 3 L of cambisol (loamic) soil. Three days after transplantation, the soil was treated with a liquid *T. harzianum* inoculum (1 × 10^7^ spores mL^−1^). The preliminary seedling assay included three treatments: (i) non-treated control (NT); (ii) treatment with a liquid spore suspension of *T. harzianum* grown in PDB and resuspended in distilled water (TT); and (iii) treatment with a suspension of *T. harzianum* grown in PDB supplemented with 6% gazpacho (TG). Ten plants per treatment were arranged in a completely randomized design. Thirty-one days after inoculation, five plants per treatment were harvested for plant height, leaf number, and dry biomass parameters. The detailed protocol and results are presented in the section 2 of Supplementary material S1.

### 2.9 Statistical analysis

All experiments involving the NaCl and gazpacho culture were conducted in triplicate, and the data are presented as mean ± standard deviation. The evaluation of the ability of *T. harzianum* to antagonize fungal phytopathogen growth was conducted with five replicates per treatment, and the results are presented as mean ± SD. Raw data are reported in section 1 of the Supplementary material S1. The preliminary seedling assay (pot trial) with tomato plants inoculated with *Trichoderma* grown in PDB and in PDB supplemented with 6% gazpacho was carried out on five plants per treatment. Raw data of preliminary tomato seedling assay (pot trial) and mean (*n* = 5) ± SD are reported in the section 2 of Supplementary material S1. For each experiment, a one-way ANOVA followed by Tukey’s HSD *post hoc* test was performed to assess statistically significant differences among treatments. All statistical analyses were performed using STATSoft Software.

## 3 Results

### 3.1 Effect of different concentrations of NaCl on *T. harzianum* in solid media

**Figure 1:**
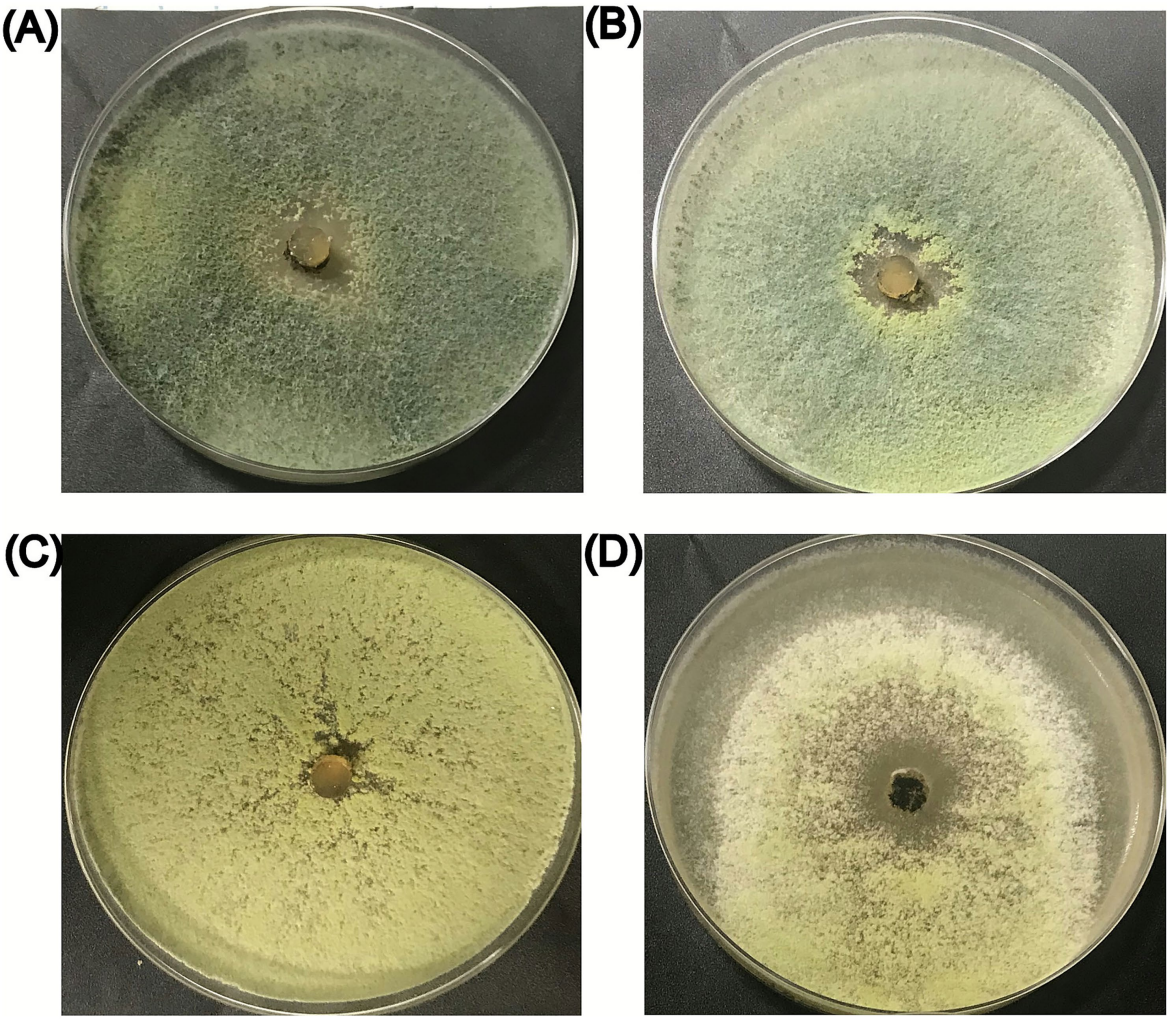
*T. harzianum* mycelium growth after 7 days post-inoculation on **(A)** PDA; **(B)** PDA + 50 mM NaCl; **(C)** PDA + 100 mM NaCl; **(D)** PDA + 150 mM NaCl.

The growth of *Trichoderma harzianum* on solid media with different NaCl concentrations was measured 7 days after inoculation. Growth significantly differed among NaCl concentrations (*p* < 0.01). The growth was maximum on PDA without NaCl and on PDA supplemented with 50 and 100 mM of NaCl, while it was significantly reduced by 15% in presence of 150 mM NaCl, compared to the PDA without NaCl (Table 1). Changes in mycelial coloration were also observed as NaCl concentration increased. Specifically, the mycelium was green on PDA without NaCl, while it gradually shifted to yellow on PDA supplemented with 50 or 100 mM NaCl. Instead, the mycelium ranged from yellow to white on PDA supplemented with 150 mM NaCl (Figure 1).

**Table 1:** Mean radial growth of *Trichoderma harzianum* on PDA supplemented with varying concentrations of NaCl.

Parameters	One-way ANOVA	PDA + NaCl			
	Df; *p-value*	0 mM	50 mM	100 mM	150 mM
Mean radial growth (mm)	3; <0.01	85.00 ± 0.00^a^	85.00 ± 0.00^a^	85.00 ± 0.00^a^	72.30 ± 0.70^b^

Mean radial growth is expressed as a mean (*n* = 3) ± standard deviation. Different letters indicate statistical differences between culture media, according to Tukey’s test (*p* ≤ 0.01).

### 3.2 Growth rate of *T. harzianum* on media containing gazpacho under SSF

The average linear growth of T*richoderma harzianum* on media with different concentrations of gazpacho varied significantly (*p* < 0.01) (Table 2). The highest growth rate was observed on PDA supplemented with 3% (v/v) gazpacho (19.25 mm/day), while the lowest occurred on PDA alone (17 mm/day). Higher linear growth on PDA supplemented with 3% (v/v) gazpacho was followed by PDA supplemented with 6% (v/v) (18.41 mm/day) and 10% (v/v) gazpacho (17.54 mm/day). These results indicated that the highest concentration of gazpacho tested in the solid medium did not improve the growth speed of *T. harzianum* compared to the PDA control.

**Table 2:** Average linear growth rate of *T. harzianum* on PDA supplemented with varying concentrations of gazpacho (0, 3, 6, and 10% (v/v)).

Parameters	One-way ANOVA	PDA + gazpacho			
	Df, *p*-value	0% (v/v)	3% (v/v)	6% (v/v)	10% (v/v)
Average linear growth rate (mm/day)	3; <0.01	17.00 ± 0.20^c^	19.20 ± 0.50^a^	18.40 ± 0.60^ab^	17.50 ± 0.30^bc^

The growth rate is expressed in millimeters per day (mm/day) as a mean (*n* = 3) ± standard deviation. Different letters indicate statistical differences between culture media, according to Tukey’s test (*p* ≤ 0.01).

**Figure 2:**
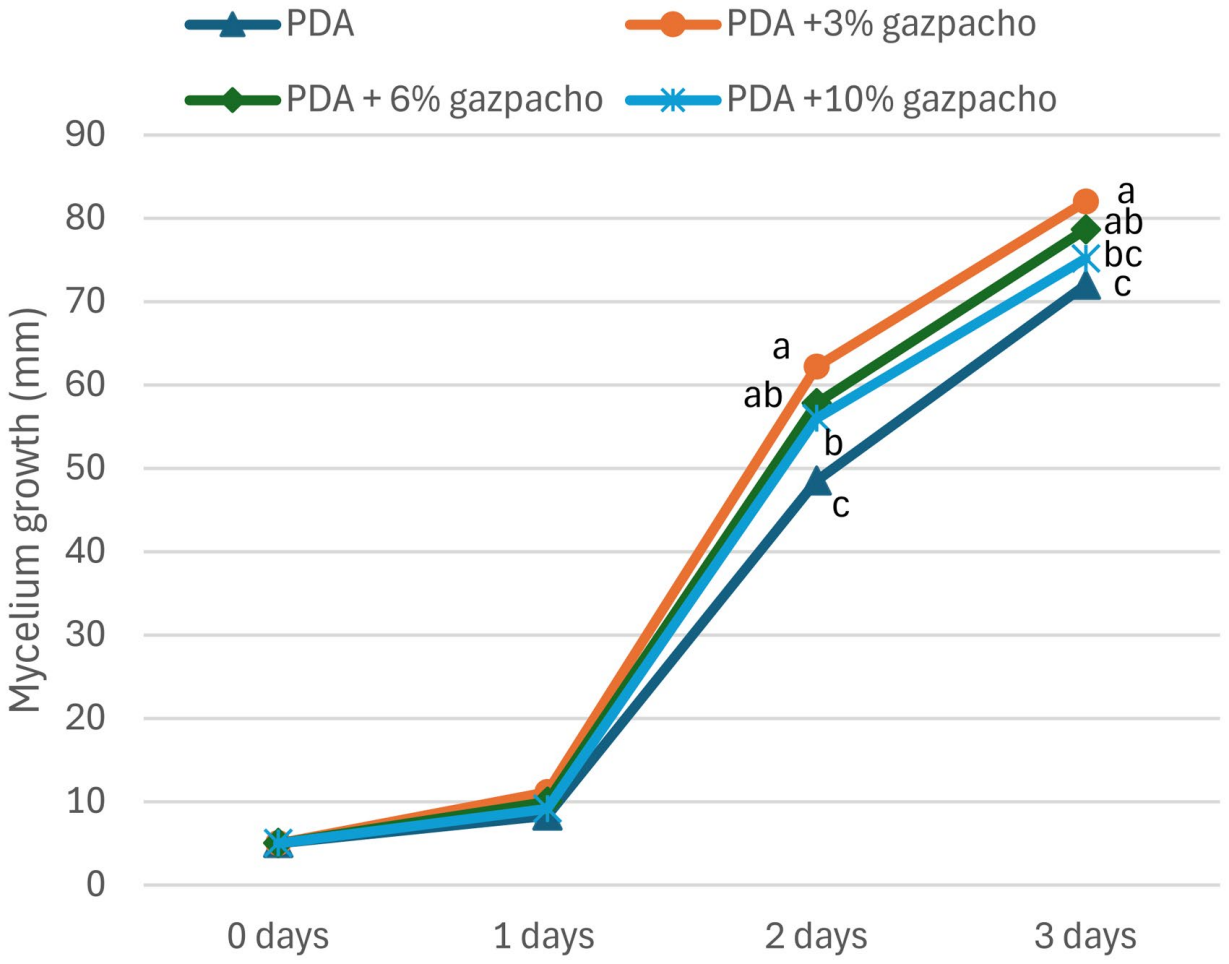
Growth trend of *Trichoderma harzianum* from day 0 to day 3 post-inoculation on PDA supplemented with varying concentration of gazpacho (0, 3, 6, and 10% (v/v)). Different letters indicate statistical differences between culture media, according to Tukey’s test (*p* ≤ 0.01).

The growth trend of *T. harzianum* on solid media supplemented with different concentrations of gazpacho was also evaluated over a 7-day period. On day 1, mycelial growth was similar across all treatments. By days 2 and 3, the highest growth was observed on PDA supplemented with 3% gazpacho, followed by 6 and 10% (v/v) gazpacho (Figure 2). The minimum growth was recorded on PDA (Figure 2). Statistically significant differences were found between PDA with 3 and 10% (v/v) gazpacho at day 3, as well as between PDA with 3% gazpacho and PDA alone at days 2 and 3. From days 4 to 7, the trend of mycelial growth was similar across all conditions, reaching maximum growth in each treatment (data not shown). Detailed mycelial growth data are provided in Supplementary Table S2.

### 3.3 Morphological characteristics of *T. harzianum* colony grown under SSF

**Figure 3:**
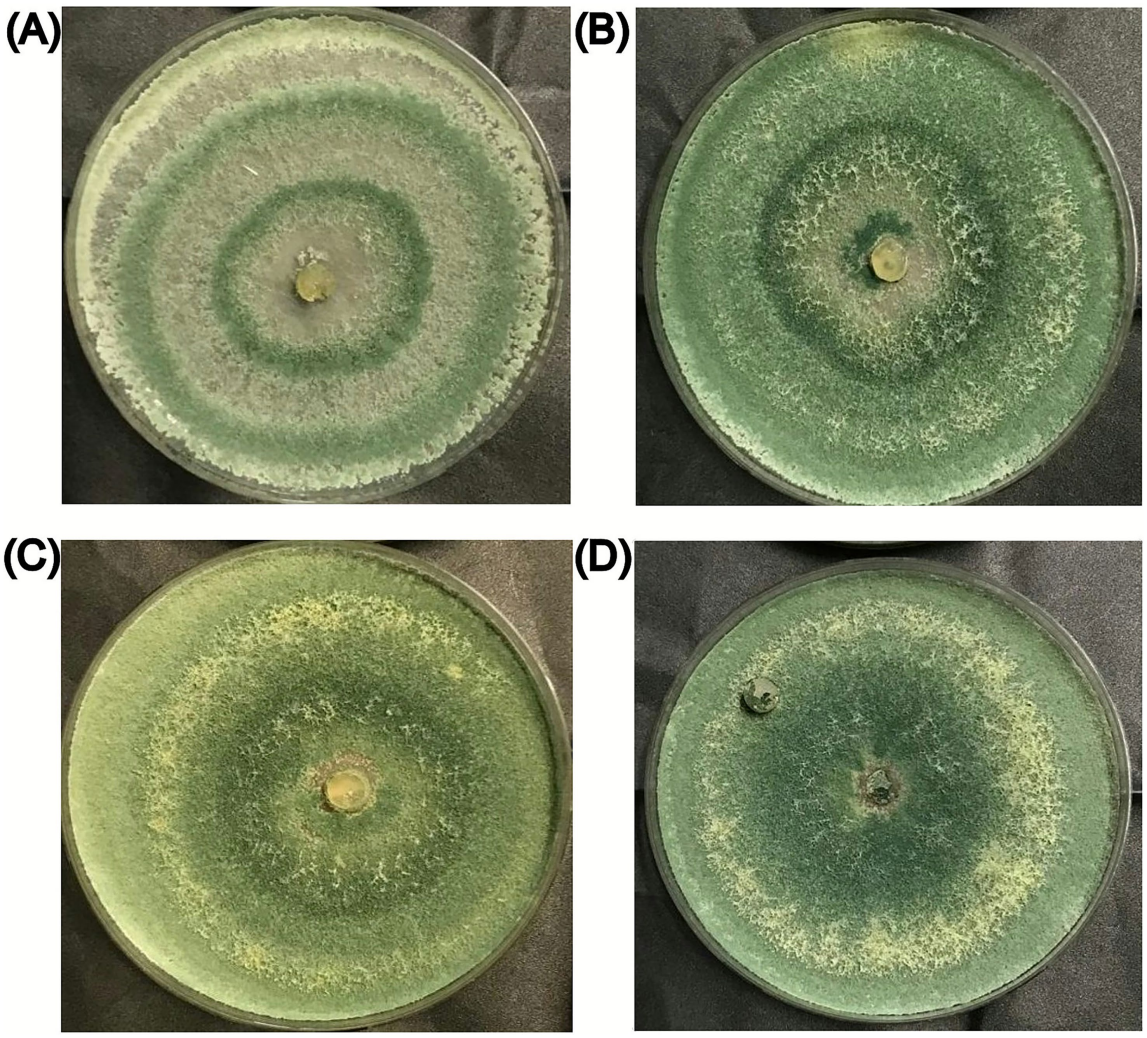
*Trichoderma harzianum* mycelial growth after 7 days of incubation on different media: **(A)** PDA; **(B)** PDA supplemented with 3% (v/v) gazpacho; **(C)** PDA supplemented with 6% (v/v) gazpacho; **(D)** PDA supplemented with 10% (v/v) gazpacho.

Colony characteristics of *Trichoderma harzianum* grown on solid media containing the different concentrations of gazpacho tested after 7 days are presented in Table 3. *Trichoderma harzianum* exhibited variation in colony morphology, such as color and surface texture, as gazpacho concentrations increased (Figure 3; Table 3). On PDA alone, *T. harzianum* exhibited a concentric ring pattern with color transitioning from white to light green (Figure 3A). With the addition of gazpacho at varying concentrations, the colony texture became increasingly compact, and the color ranged from green to dark green (Figures 3B–D).

**Table 3:** Mycelial characteristics of *Trichoderma harzianum* grown under SSF on media on PDA supplemented with varying concentrations of gazpacho (0, 3, 6, and 10% (v/v)).

Growth media	Color	Surface	Margin	Surface texture
PDA	White to light green	Upper	Regular	Concentric ring, loose compact
PDA + 3% (v/v) gazpacho	Light green to green	Upper	Regular	Compact
PDA + 6% (v/v) gazpacho	Green to dark green	Upper	Regular	Moderately compact
PDA + 10% (v/v) gazpacho	Dark green	Upper	Regular	Highly compact

### 3.4 Growth of the *T. harzianum* strain at different concentrations of gazpacho under submerged-state fermentation (SmF)

The biomass of *Trichoderma harzianum* increased significantly (*p* < 0.01) with the increasing concentrations of gazpacho. Specifically, after 7 days of growth in submerged fermentation condition, the biomass reached 5.0 ± 0.2 g·L^−1^ at 3% (v/v) gazpacho, 6.0 ± 0.6 g·L^−1^ at 6%, and 8.0 ± 0.5 g·L^−1^ at 10%, corresponding to 19, 32, and 87% increases, respectively, compared with the control (4.0 ± 0.6 g·L^−1^) (Table 4). Biomass was significantly different between PDB alone and PDB + 6% (v/v) gazpacho and PDB alone and PDB + 10% (v/v) gazpacho. Instead, it was not significantly different between PDB alone and PDB + 3% (v/v) gazpacho and between PDB + 3% (v/v) gazpacho and PDB + 6% (v/v) gazpacho, as shown in Table 4. In addition, spore production also increased gradually from PDB without gazpacho to PDB supplemented with 6% (v/v) gazpacho, ranging from 1.70 × 10^7^ to 4.10 × 10^7^ spores mL^−1^. However, despite the highest biomass was observed at 10% gazpacho, spore production was significantly reduced (0.80 × 10^7^ spore mL^−1^), compared to the other media. To optimize both biomass and spore yield, subsequent analyses were focused on 3 and 6% (v/v) gazpacho—concentrations, which resulted in improvements of both parameters. The effect of NaCl on mycelial growth and spore production of *T. harzianum* under SmF.

**Table 4:** Mycelial biomass and spore production of *T. harzianum* grown under SmF using PDB supplemented with different concentrations of gazpacho (0, 3, 6, and 10% (v/v)).

Parameters	One-way ANOVA	PDB + gazpacho			
	df; *p*-value	0% (v/v)	3% (v/v)	6% (v/v)	10% (v/v)
Dry weight mycelium (g·L^−1^)	3; <0.01	4.00 ± 0.6^c^	5.00 ± 0.2^bc^	6.00 ± 0.6^b^	8.00 ± 0.5^a^
Number of spores (10^7^ spores mL^−1^)	3; <0.01	1.70 ± 0.06^b^	1.50 ± 0.02^c^	4.10 ± 0.10^a^	0.80 ± 0.01^d^
pH	3; <0.01	4.80 ± 0.00^a^	4.70 ± 0.03^b^	4.60 ± 0.04^c^	4.40 ± 0.04^d^

Mycelial biomass is expressed as dry weight mycelium (g·L^−1^), and spore concentration is given in spore mL^−1^. Data are reported as mean (*n* = 3) ± standard deviation. *p*-value indicates one-way ANOVA test. Different letters indicate statistical differences between culture media, according to Tukey’s test (*p* ≤ 0.01).

**Figure 4:**
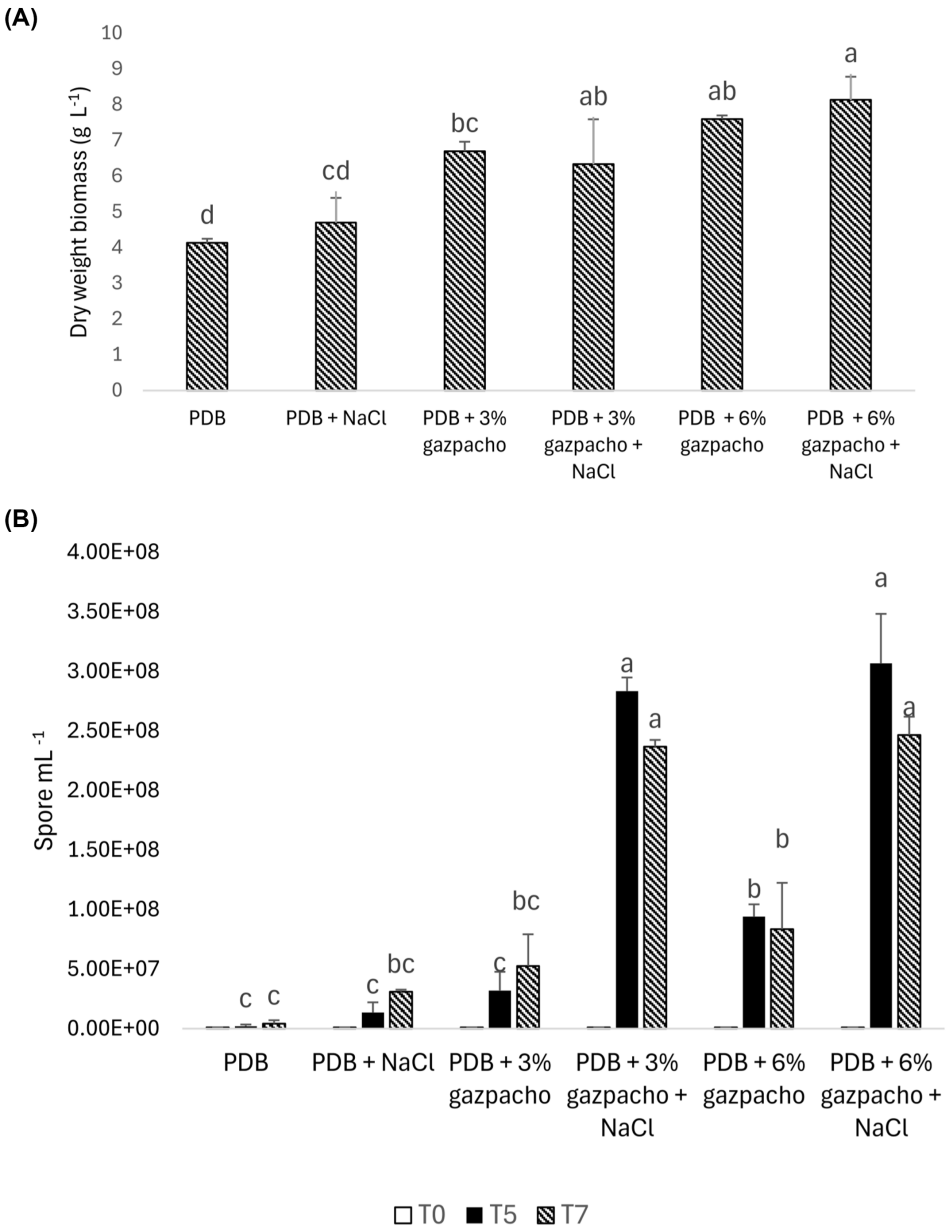
*Trichoderma harzianum* growth in submerged fermentation with and without the addition of 3 and 6% gazpacho and 100 mM NaCl. **(A)** Dry biomass weight after 7 days of incubation. **(B)** Spore concentration measured at 0, 5, and 7 days. Different letters indicate statistical differences between culture media for each time point, according to Tukey’s test (*p* ≤ 0.01).

The effect of 100 mM NaCl on mycelial growth and spore production of *Trichoderma harzianum* grown in liquid medium containing 3 and 6% (v/v) gazpacho is shown in Figure 4. At 7 days post-inoculation, the addition of gazpacho to the culture medium led to a significant increase in the fungal biomass of *T. harzianum* (*p* < 0.01) (Figure 4A). Mycelial biomass in PDB alone was significantly lower than in all gazpacho-supplemented cultures. The addition of gazpacho to the liquid medium significantly enhanced *Trichoderma* biomass by 63.41% at 3% (v/v) gazpacho and by 84.01% at 6% (v/v), relative to the control. Although biomass was higher at 6% than at 3% (v/v) gazpacho, the difference between these two concentrations was not statistically significant. Additionally, the presence of NaCl in the liquid culture did not result in statistically significant differences in fungal biomass compared to the corresponding salt-free treatments.

In contrast to biomass, spore production observed at different days after inoculation increased in presence of gazpacho and further increased in presence of both gazpacho and NaCl (*p* < 0.01). Specifically, in PDB medium supplemented with 3% (v/v) gazpacho, spore production reached 3.16 × 10^7^ spore mL^−1^ after 5 days, an increase of 16 times compared to the control, and further increased to 5.26 × 10^7^ spore mL^−1^ at 7 days, corresponding to 12 times increase. At 6% (v/v) gazpacho, spore production increased to 9.40 × 10^7^ spores mL^−1^ after 5 days (a 29-time increase) and to 7.57 × 10^7^ spores mL^−1^ after 7 days (a 19.6-time increase) compared to the control. When NaCl was added in the liquid culture supplemented with gazpacho, the spores further increased, with the highest concentration observed in the medium containing both 6% (v/v) gazpacho and NaCl. The statistical differences in spore concentration between treatments for each time point are reported in Figure 4B.

### 3.5 Ability of *T. harzianum* to solubilize rock phosphate under SmF containing gazpacho

*Trichoderma harzianum* was able to solubilize the rock phosphate supplemented in the liquid fermentation media with 6% (v/v) of gazpacho (*p* < 0.01) (Table 5). The concentration of solubilized phosphorus (P) measured in the fermentation media supplemented with gazpacho, in the absence of RP, was lower than when gazpacho was added. These values of soluble P could not be explained by the microbial action and most likely corresponded to the soluble P fraction of gazpacho. The pH of the liquid media increased when RP was added to the fermentation media with gazpacho, due to the slight neutralization (*p* < 0.01).

**Table 5:** PH and phosphate concentration solubilized in SmF by *T. harzianum* with gazpacho and rock phosphate at the end of the liquid fermentation.

Parameters	One-way ANOVA	PDB + gazpacho		
	df; *p-value*	0% (v/v)	6% (v/v)	6% (v/v) + rock phosphate (3 g·L^−1^)
pH	2; <0.01	4.90 ± 0.90^b^	5.20 ± 0.15^b^	6.10 ± 0.21^ab^
Phosphate solubilized	2; <0.01	0.00^b^	18.00 ± 2.00^b^	126.00 ± 14.00^a^

Data are reported as a mean (*n* = 3) ± standard deviation. Different letters indicate statistical differences between culture media, according to Tukey’s test (*p* ≤ 0.01).

### 3.6 Ability of *T. harzianum* to antagonize the fungal phytopathogens growth

**Figure 5:**
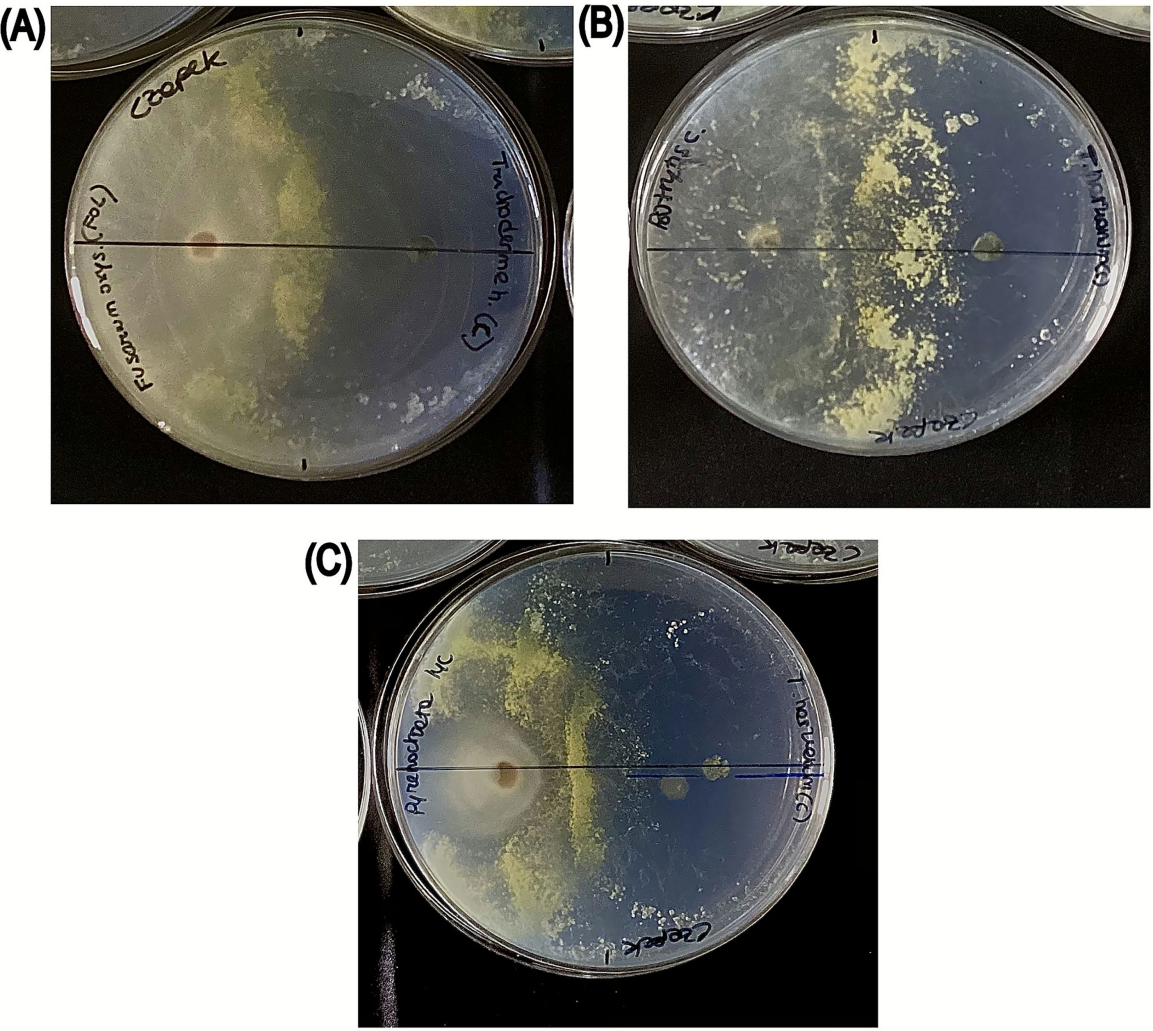
Dual culture assay of *T. harzianum* against fungal phytopathogens. **(A)** *F. oxysporum* **(B)** *B. cinerea* **(C)** *P. lycopersici*. In each dual-culture plate, the fungal pathogen is inoculated on the left side and *T. harzianum* on the right side.

Dual culture results showed that *Trichoderma harzianum* limited the growth of all fungal phytopathogens tested with different growth inhibition patterns as shown in Figure 5. The highest inhibition rate was observed against *B. cinerea* (78.40%), followed by *P. lycopersici* (76.00%) and *F. oxysporum* (74.10%) (Table 5). However, the differences in inhibition percentages among the pathogens were not statistically significant. Furthermore, the inhibitory behavior of *T. harzianum* differed for the three pathogenic fungi tested, as reported in Figure 5: the growth of *P. lycopersici* and *F. oxysporum* was inhibited at the point of interaction (Figures 5A–C), and their mycelium was forced to grow toward the opposite side of the Petri dish, away from *T. harzianum*. While for the dual culture *T. harzianum* - *B. cinerea* the growing mycelium produced by both colonies approach each other until they are almost in contact, leaving a well-marked, narrow, unoccupied space between them (Figure 5B).

## 4 Discussion

The major concern in developing technological schemes and commercial production of bioinoculants is to ensure the specific nutritional and process conditions for their adequate growth and spores formation. Many studies have investigated cost-effective production of *Trichoderma* biomass and spores using liquid- and solid-state fermentation methods, typically employing inexpensive media derived mainly from solid or liquid agro-industrial residues (Tewari and Bhanu, 2004; Amaresan et al., 2022). Among agro-industrial residues, tomato by-products can be considered a significant portion of the waste generated annually by the world’s most widespread industries (Almeida et al., 2024). These by-products are mainly used for recovering bioactive compounds, such as carotenoid (lycopene, β-carotene) and phenolic compounds for food, pharmaceutical, and cosmetic sectors and for production of biogas by anaerobic digestion. However, to our knowledge, no studies were focused on the use of any tomato by-product for mass multiplication of microorganisms (Simon, 2011; Eslami et al., 2023). Thus, the present study was planned as a preliminary assessment of the use of gazpacho, as a tomato byproduct, as supplement in the culture media for the mass multiplication of *T. harzianum* and to explore its multifunctional potential for application in tomato cultivation.

### 4.1 Effect of different concentrations of gazpacho on *T. harzianum* growth

The main factors that influence the mass production of fungi include temperature, pH, nutrient composition of substrate and moisture content (Sachdev et al., 2018). In many studies, growth and media composition requirements for biomass and spore production of *Trichoderma* on agricultural by-products and wastes have been optimized by applying various optimization models. For example, sugarcane bagasse, corn cobs, rice straw, wheat straw, peat-bran, and groundnut shell were found to be good media under SSF (Mulatu et al., 2021). In our study, the addition of gazpacho on the media increased the biomass and spore production under solid and liquid submerged fermentation. Notably, concentrations of 3 and 6% (v/v) gazpacho significantly enhanced both biomass accumulation (5.0, 6.0 g·L^−1^), corresponding to 19 and 32%, respectively, and spore production, with the highest sporulation obtained at 6% (v/v) gazpacho (4.10 × 10^7^ spore mL^−1^), compared to the growth on the media without gazpacho (4 g·L^−1^, 1.7 × 10^7^ spore mL^−1^). This increase can be likely attributed to the improved availability of nutrients in the tomato by-product, composed by carbon source as cellulose and glucose, essential for providing energy to growth, while increased sporulation can be explained by minerals, such as nickel, copper, iron, manganese, potassium, zinc, magnesium, molybdenum, and boron, essential for sporulation (Ma Verde Méndez et al., 2008; Borkertas et al., 2025). In contrast, the highest gazpacho concentration (10% v/v) increased biomass by 87% but reduced spore production by 52.9% as compared to the control. The decreased sporulation may be imputed to the acidity of gazpacho, that decreased the medium pH down to 4.4, which was lower than the media containing the lower concentrations. In fact, it has been reported that growth and sporulation of all different species of *Trichodema* decreased significantly with pH below 4.6 or above 7.6 (Zehra et al., 2017). However, the effect of pH on sporulation remains tentative because no specific experiments were conducted to identify the underlying mechanisms. In fact, additional components present in gazpacho, such as organic acids, phenolics compounds may also influence the sporulation. Thus, the effect of different concentrations of these compounds, as well as other factors as osmotic pressure, temperature, pH, moisture on *Trichoderma* growth should be evaluated for product formulation. Moreover, it is important to keep in mind that tomato by-products are highly variable, depending on factors such as cultivar., type of residue, and season. These factors influence taste-active compounds—including dry matter, sugars, organic acids, carotenoids, volatiles—as well as antioxidant phytochemicals such as ascorbic acid, phenols, and sulfur-reducing compounds, all of which determine the organoleptic and nutraceutical properties of tomato fruit. Industrial processing further affects the preservation and availability of these bioactive compounds (Motilva et al., 2022; Burato et al., 2025). As a result, tomato-based products such as gazpacho, sauces, and purées do not have uniform compositions across different batches. Consequently, in our study, the use of a single batch prevents us from generalizing the results to all products generated by the industry. Despite this limitation, our preliminary results may hinder the use of gazpacho or other tomato by-products as complete alternative substrates but demonstrate that they could be successfully adopted as supplements to standard growth media. For instance, the integration of waste substrates into culture media was supported for other agro-wastes, such as sugarcane molasses–supplemented medium, where biomass production was higher when molasses was used as a supplement rather than as a full replacement of the standard medium (Machado et al., 2018). However, our study is still a result obtained in the screening phase and requires further optimization and formulation studies. For this reason, further studies are needed to develop experimental protocols that evaluate the effects of environmental factors on growth of *T. harzianum*. For example, a first experiment might be conducted using a response Surface Methodology (RSM) approach with a 3-level factorial design in which the percentage of gazpacho (3, 6, 10%), pH (3.5, 4.5, 5.5 and 6.5) and temperature (25, 30, 35 °C), would be evaluated to model their combined effects on *Trichoderma* growth and sporulation (dry biomass, g·L^−1^; spores, spore·mL^−1^). This approach would allow to describe factor interactions and identify optimal conditions, thereby providing valuable guidance for process optimization. Moreover, to reduce the variability of the gazpacho, mitigation strategies should include the characterization of raw materials using quality-control (QC) metrics such as soluble solids (°Brix), pH, and total nitrogen. Process-related measures can also help, such as batch mixing—e.g., blending high-°Brix concentrates with low-°Brix batches to achieve the desired °Brix and pH targets. Additional pre-treatment strategies may include using a fixed hot-break temperature (85–95 °C) to standardize enzyme inactivation and viscosity, as well as removing water from low-solids fruit to increase °Brix before mixing.

To contextualize our results, optimized *Trichoderma* production systems typically achieve conidial yields on the order of 10^7^–10^8^ spores·mL^−1^ in submerged fermentation (SmF) and 10^8^–10^9^ spores·g^−1^ in solid-state fermentation (SSF), depending on strain, substrate, and process conditions (Vassileva N. et al., 2009; Abuhena et al., 2022; Kumar V. et al., 2023). In the present study, the highest spore concentration was observed in PDB supplemented with 6% (v/v) gazpacho, reaching 4.10 × 10^7^ CFU·mL^−1^, while PDB alone produced 1.70 × 10^7^ CFU·mL^−1^. These values fall within the lower range of typical yields reported for optimized SmF cultures, consistent with the exploratory, screening-level nature of this work. Recent studies demonstrate that agro-industrial wastes and food by-products can be successfully exploited as substrates for *Trichoderma* cultivation, enabling substantial biomass and spore production. For example, solid-state fermentation of *Trichoderma* spp. using agricultural digestate combined with food residues achieved high sporulation levels, demonstrating the potential of non-conventional substrates (Alias et al., 2022). Similarly, a 2024 review highlights the increasing use of agro-food residues for *Trichoderma* cultivation, emphasizing the feasibility of integrating waste valorization into industrially relevant processes (Lima et al., 2024). Further examples include SSF on green waste for biostimulant production (2023), and production using tea processing residues achieving ∼10^9^ CFU·g^−1^, illustrating that optimized waste-derived substrates can reach yields comparable to traditional media(Meng et al., 2024). Given these precedents and the stimulatory effect of gazpacho observed in our experiments, substantial improvements could be expected following systematic optimization. DOE- and RSM-based studies have reported yield increases of one to two orders of magnitude after optimizing key variables such as carbon-to-nitrogen ratio, substrate composition, moisture, and incubation parameters (Sachdev et al., 2018; Sala et al., 2019; Abuhena et al., 2022). Therefore, these results should be regarded as a preliminary proof-of-concept, providing a foundation for further optimization, scale-up, and formulation research necessary for industrial translation (Vassileva et al., 2010; Kumar V. et al., 2023; Lima et al., 2024).

### 4.2 Evaluation of multifunctional activities of *T. harzianum*

The evaluation of the multifunctional potential of *T. harzianum* has also been evaluated. The antagonistic activity of *T. harzianum* strain against the growth of *B. cinerea, F. oxysporum* and *P. lycopersici*, three fungal pathogens of the tomato plant, was examined in the present study. The dual culture assay showed that *T. harzianum* exhibited antagonistic activity against the selected fungal pathogens, inhibiting >70% of mycelial growth. Regarding the inhibition rate, *B. cinerea* displayed the highest value of 78.40%, followed by *P. lycopersici* (76.00%) and *F. oxysporum* (74.00%). The inhibition of *F. oxysporum* was in line with other studies which screened the ability of *T. harzianum* T16 and T23 to inhibit the growth of different *F. oxysporum* isolates (Altinok and Erdoğan, 2015). Similar findings have been reported by other studies on other pathogenic fungi (Álvarez and Ramírez, 2004; Idris et al., 2007; Hajieghrari et al., 2008). Also, the inhibition of *P. lycopersici* is consistent with findings in early studies where *Trichoderma* spp. inhibited the growth of *P. lycopersici* by 20–100% in dual culture (Sánchez et al., 2013). *Trichoderma* species are known to produce a number of antibiotics as well as some cell wall degrading enzymes like chitinase and glucanase hydrolytic enzymes which are closely related to mycoparasitism (Elad, 2000). However, they seem to have influenced its mechanism of action. In fact, according to Porter, inhibitory behavior of the *in vitro* growth on a solid medium of one fungus against another can be categorized into (a) mutually slight inhibition, (b) growth around, (c) overgrowing, and (d) inhibition at a distance (Porter, 1924). In this work, a different type of inhibitory behavior was observed, which can be categorized as growth around for dual culture against *P. lycopersici* and *F. oxysporum* and mutual slight inhibition for dual culture against *B. cinerea*. These findings underscore the variability in *T. harzianum’s* biocontrol efficacy, which is influenced by the specific fungal species it encounters (Dourou and La Porta, 2023). This hypothesis is confirmed by the fact that antagonistic activity is not limited to different genera, but these authors have observed that *Trichoderma* also exhibits antagonistic activity in dual culture with isolates of the same species. The possible reason resides in the mechanisms developed by fungi to accurately recognize themselves and not harm cells belonging to the same species or genus (Porter, 1924). In our study, a formation of inhibition contact was found against all fungi tested. This could be explained on the basis of the production of volatile and non-volatile metabolites (such as terpenes, pyrones, polyketides, etc), as well as the production of hydrolytic enzymes by *T. harzianum* (Zehra et al., 2017). However, further experiments will be necessary to allow us to understand the mechanisms of action used by *T. harzianum*.

Furthermore, *T. harzianum* was able to solubilize rock phosphate on media containing gazpacho in submerged condition indicating that this material was a good substrate for phosphate solubilization. The solubilization of RP on media containing agro industrial waste was firstly tested by Vassileva et al. in different experiments with wastes derived from the sugar-producing and olive oil industries (Vassileva et al., 2006; Vassileva M. et al., 2009). The possible mechanisms consist into the release of organic compounds by *T. harzianum*, that make free inorganic phosphate for solubilization. In fact, organic acids are excellent P-solubilizing agents and, therefore, bio-treatment of low-grade RP by fungal fermetation can be accepted as an attractive approach within microbially mediated solubilization.

Salt stress is another important abiotic factor that highly impacts the growth of *T. harzianum*. In our study, *T. harzianum* was able to growth on media containing up to 100 mM of NaCl, while the growth was decreased at 150 mM NaCl. Considering these findings, we also tested the effect of NaCl on *T. harzianum* grown on media containing 3% (v/v) and 6% (v/v) of gazpacho. The biomass of *T. harzianum* grown on media containing 3% (v/v) and 6% (v/v) of gazpacho in presence of NaCl, in submerged condition was not affected by 100 mM of sodium chloride tested. Instead, a higher sporulation of *T. harzianum* in presence of NaCl was obtained. The highest sporulation was obtained when in the liquid medium was present 6% (v/v) gazpacho and NaCl. Similar to our results, other studies revelead that of the 45 *T. harzianum* isolates, only five isolates were salinity tolerant and able to growth and sporulate up to 240 mM (Rawat et al., 2013). However, other researchers demostrated that the salt has or not a significant negative impact on the mycelial growth and sporulation of *T. harzianum* isolates (Gal-Hemed et al., 2011; Kumar et al., 2017). These differences may be due to the different source of isolation of this fungus and due to the strain differences. Different mechanisms by which microbes tolerate the salt stress were explained, such as the accumulation of various inorganic ions intracellularly to create an equilibrium in the salt concentration, synthesis of organic compatible solute and enzymatic adaptation (Arthi et al., 2024). Characterization of *T. harzianum* salt tolerant mutants done by Mohamed and Haggag, showed that the mutants have reinforced contents of proline and hydroxyproline, amminoacids that act as an compatible solute for maintaing the osmoregulation from oxydative damage (Mohamed and Haggag, 2006).

## 5 Conclusions and perspectives

This study represents a laboratory scale feasibility assessment of using commercial gazpacho residue as a supplemental substrate for *T. harzianum*. This preliminary study demonstrates that tomato-derived by-products, exemplified by commercial gazpacho, can be effectively used as cost-efficient supplements in the culture media for *T. harzianum* mass production. These findings confirm that tomato residues can improve fungal yield and reduce production costs without compromising performance, supporting a circular bioeconomy approach to bioinoculant manufacturing. However, these characteristics should be further examined under different fermentation processes to establish well-defined conditions for metabolic control, including the evaluation of the shelf-life of the product. Moreover, other parameters such as pH, temperature and humidity as well as different nutrients should be evaluated in future studies as well as their interactions and their effect on the fungal yield, to optimize the production process toward large-scale production systems. Future work will address substrate standardization, optimization, bioreactor validation, and formulation/shelflife testing to evaluate industrial translation.

Our results demonstrate that *T. harzianum* exhibited strong antagonistic activity against major tomato pathogens, including *Botrytis cinerea*, *Fusarium oxysporum*, and *Pyrenochaeta lycopersici*, with inhibition rates exceeding 70%. The fungus also displayed phosphate-solubilizing ability and tolerance to moderate salt stress (up to 100 mM NaCl), highlighting its multifunctional potential as a biocontrol agent and biofertilizer in sustainable tomato cultivation as well as its broader applicability in environmental, biotechnological, and food-processing industries. These findings emphasize the importance of simultaneously investigating multiple metabolic traits of soil microorganisms (Vassileva et al., 2010).

Finally, a validation of the efficacy of *T. harzianum* grown on media supplemented with 6% (v/v) gazpacho should be addressed under real conditions. Actually, a pot-scale experiment is being conducted using tomato seedlings in greenhouse. Preliminary results indicate that the height of inoculated seelings was, on average, 26% higher than that of non-inoculated plants, thus providing encouraging indications of the potential beneficial effects of the bioinoculum (Supplementary Table S6; Supplementary Figure S1).

Overall, these results emphasize the importance of exploiting agro-industrial by-products as nutrient supplements to promote cost-effective and environmentally friendly bioinoculant production. The ability of *T. harzianum* to thrive under saline conditions and enhance plant health further reinforces its value as a versatile tool in sustainable agriculture. Future work should optimize fermentation parameters and expand testing to other agro-industrial residues to develop robust, scalable production systems for multifunctional microbial bioinoculants.

## Data Availability

The raw data presented in the study are included in the Supplementary material. Further inquiries can be directed to the corresponding author.
