# Supplementary material for: Evaluation of tomato based agro-industrial byproducts as substrates for Trichoderma harzianum cultivation and bioinoculant potential
Source: Front Microbiol. 2026 Jan 16;16:1713960. doi: 10.3389/fmicb.2025.1713960 (PMC12858247; doi:10.3389/fmicb.2025.1713960)
Supplement: Supplementary file 1 [file Data_Sheet_1.ZIP › supplementary materials S1/Supplementary material S1.docx]

**Supplementary material S1**

The supplementary materials include tables reporting the raw data from experiments assessing the tolerance of  *T. harzianum* grown at different NaCl concentrations, the evaluation of biomass and spore production under solid and submerged state fermentation supplemented with different gazpacho concentrations (1), and the effect of *T. harzianum*  grown on PDB supplemented with 6% gazpacho in a tomato seedling–soil system under pot-trial conditions (2).

1. **Growth of *T. harzianum* under different NaCl and gazpacho concentrations.**

**Table S1**. Growth of *Trichoderma harzianum* on PDA supplemented with varying concentrations of NaCl (0 mM – control, 50 mM, 100, 150 mM NaCl). The number of the replicates is indicated as R1, R2, and R3.

| **Medium** | **Mycelium growth (mm)** |
| --- | --- |
| PDA_R1 | 85.00 |
| PDA_R2 | 84.90 |
| PDA_R3 | 84.98 |
| PDA + 50 mM NaC_R1 | 85.00 |
| PDA + 50 mM NaCl_R2 | 84.90 |
| PDA + 50 mM NaCl_R3 | 84.90 |
| PDA +100 mM NaCl_R1 | 85.00 |
| PDA +100 mM NaCl_R2 | 84.80 |
| PDA +100 mM NaCl_R3 | 84.90 |
| PDA +150 mM NaCl_R1 | 72.50 |
| PDA +150 mM NaCl_R2 | 71.50 |
| PDA +150 mM NaCl_R3 | 73.00 |

**Table S2.** Mycelium growth of *T. harzianum* on PDA supplemented with varying concentrations of gazpacho (0%, 3%, 6%, and 10%) from day 0 to day 7. The number of the replicates is indicated as R1, R2, and R3.

|  | **Mycelium growth (mm)** | | | | |
| --- | --- | --- | --- | --- | --- |
| **Medium** | **Day 1** | **Day 2** | **Day 3** | **Day 4** | **Day 7** |
| PDA_R1 | 8.00 | 49.00 | 72.50 | 72.50 | 85.00 |
| PDA_R2 | 8.09 | 46.50 | 72.50 | 74.50 | 85.00 |
| PDA_R3 | 9.00 | 50.00 | 71.00 | 72.00 | 85.00 |
| PDA + 3%_gazpacho_R1 | 11.00 | 60.00 | 80.00 | 85.00 | 85.00 |
| PDA + 3%_gazpacho_R2 | 12.50 | 65.00 | 82.50 | 85.00 | 85.00 |
| PDA + 3%_gazpacho_R3 | 10.00 | 61.50 | 83.50 | 85.00 | 85.00 |
| PDA + 6%_gazpacho_R1 | 10.00 | 61.50 | 76.00 | 85.00 | 85.00 |
| PDA + 6%_gazpacho_R2 | 9.00 | 56.50 | 80.00 | 85.00 | 85.00 |
| PDA + 6%_gazpacho_R3 | 11.00 | 55.50 | 80.00 | 85.00 | 85.00 |
| PDA + 10% gazpacho_R1 | 9.00 | 55.00 | 76.50 | 85.00 | 85.00 |
| PDA + 10% gazpacho_R2 | 10.00 | 55.50 | 75.00 | 85.00 | 85.00 |
| PDA + 10% gazpacho_R3 | 8.50 | 57.50 | 74.00 | 85.00 | 85.00 |

**Table S3**. Mycelial biomass, spore production of *T. harzianum* grown under SmF using PDB supplemented with different concentrations of gazpacho (0%, 3%, 6%, and 10%) at the end of SmF. Initial pH of medium is reported in the table. The number of the replicates is indicated as R1, R2, and R3.

| **Medium** | **Dry weight micelium (g L^-1^ )** | **Spore mL^-1^** | **pH** |
| --- | --- | --- | --- |
| PDB_R1 | 3.61 | 1.80E+07 | 4.80 |
| PDB_R2 | 4.61 | 1.81E+07 | 4.76 |
| PDB_R3 | 4.74 | 1.70E+07 | 4.77 |
| PDB + 3% gazpacho_R1 | 5.15 | 1.50E+07 | 4.70 |
| PDB+ 3% gazpacho_R2 | 5.33 | 1.51E+07 | 4.72 |
| PDB + 3% gazpacho_R3 | 4.98 | 1.53E+07 | 4.68 |
| PDB + 6% gazpacho_R1 | 5.88 | 4.10E+07 | 4.57 |
| PDB +6% gazpacho_R2 | 6.18 | 4.30E+07 | 4.63 |
| PDB + 6% gazpacho_R3 | 5.05 | 4.23E+07 | 4.60 |
| PDB + 10% gazpacho_R1 | 7.54 | 8.40E+06 | 4.40 |
| PDB + 10% gazpacho_R2 | 8.46 | 8.50E+06 | 4.36 |
| PDB + 10% gazpacho_R3 | 8.27 | 8.30E+06 | 4.44 |

**Table S4**. Growth of *T. harzianum* in submerged fermentation with and without the addition of 3% and 6% gazpacho and 100 mM NaCl. Dry weight mycelium after 7 days of incubation, and spore concentration measured at 0 (T0), 5 (T5), and 7 (T7) days are reported in the table. The number of the replicates is indicated as R1, R2, and R3.

| **Medium** | **Dry weight mycelium**  **(g L^-1^)** | **Spore mL^-1^** | | |
| --- | --- | --- | --- | --- |
|  |  | **T0** | **T5** | **T7** |
| PDB_R1 | 4.00 | 2.15E+08 | 6.30E+05 | 2.30E+06 |
| PDB_R2 | 4.20 | 2.15E+08 | 1.70E+06 | 3.00E+06 |
| PDB_R3 | 4.20 | 2.15E+08 | 3.60E+06 | 7.50E+06 |
| PDB + NaCl_R1 | 4.30 | 2.15E+08 | 1.90E+07 | 3.20E+07 |
| PDB + NaCl_R2 | 4.30 | 2.15E+08 | 3.80E+06 | 2.90E+07 |
| PDB + NaCl_R3 | 5.50 | 2.15E+08 | 1.80E+07 | 3.20E+07 |
| PDB + 3% gazpacho_R1 | 6.80 | 2.15E+08 | 2.90E+07 | 2.50E+07 |
| PDB+ 3% gazpacho_R2 | 6.40 | 2.15E+08 | 1.80E+07 | 7.80E+07 |
| PDB + 3% gazpacho_R3 | 6.90 | 2.15E+08 | 4.90E+07 | 5.50E+07 |
| PDB + 3% gazpacho + NaCl_R1 | 5.00 | 2.15E+08 | 2.70E+08 | 2.40E+08 |
| PDB + 3% gazpacho + NaCl_R2 | 6.50 | 2.15E+08 | 2.90E+08 | 2.40E+08 |
| PDB + 3% gazpacho + NaCl_R3 | 7.50 | 2.15E+08 | 2.90E+08 | 2.30E+08 |
| PDB + 6% gazpacho_R1 | 7.50 | 2.15E+08 | 1.00E+08 | 4.70E+07 |
| PDB +6% gazpacho_R2 | 7.70 | 2.15E+08 | 8.20E+07 | 1.20E+08 |
| PDB + 6% gazpacho_R3 | 7.60 | 2.15E+08 | 1.00E+08 | 6.00E+07 |
| PDB + 6% gazpacho + NaCl_R1 | 8.10 | 2.15E+08 | 3.40E+08 | 2.50E+08 |
| PDB + 6% gazpacho + NaCl_R2 | 7.50 | 2.15E+08 | 2.60E+08 | 2.60E+08 |
| PDB + 6% gazpacho + NaCl_R3 | 8.80 | 2.15E+08 | 3.20E+08 | 2.30E+08 |

**Table S5**. pH and phosphate concentration solubilised in SmF by *T. harzianum* with gazpacho and rock phosphate (RP) at the end of the SmF. The number of the replicates is indicated as R1, R2, and R3.

| **Medium** | **pH** | **Phosphate (P) solubilized** |
| --- | --- | --- |
| PDB_R1 | 4.69 | 0.00 |
| PDB_R2 | 5.07 | 0.00 |
| PDB_R3 | 4.88 | 0.00 |
| PDB + 6% gazpacho_R1 | 4.60 | 20.00 |
| PDB + 6 %gazpacho_R2 | 4.70 | 18.00 |
| PDB + 6 % gazpacho_R3 | 4.68 | 16.00 |
| PDB + 6 % gazpacho + RP (3g L^-1^) _R1 | 6.26 | 120.00 |
| PDB + 6 % gazpacho + RP (3g L^-1^) _R2 | 6.10 | 116.00 |
| PDB + 6 % gazpacho + RP (3g L^-1^) _R3 | 5.84 | 142.00 |

1. **Tomato seedlings-soil experiment in pot scale condition**

**Methods S1**. Inoculation of *T.* *harzianum* as plant growth promoting bioinoculant of tomato seedlings in a pot experiment.

The trial started in September 2025. Tomato seedlings (cv. Cuore di bue) (Esposito, A., Scala, V., Vitali, F., Beccaccioli, M., Reverberi, M., Valboa, G., ... & Mocali, S. (2025). Exploring the Root-Associated Bacterial Community of Tomato Plants in Response to Salt Stress. *Agriculture; Basel*, *15*(6)), of uniform size (15 cm in height, 10 leaves) were transplanted into pots, one plant per pot. The pots had an 18 cm diameter, a volume of 4 L, and were filled with 3 L of Cambisol (Loamic) soil with the following chemical and physical properties: 1.11% organic carbon, 0.09% total nitrogen, sandy clay loam texture, and pH 8.55. The tomato seedling assay was conducted in a greenhouse under natural conditions, without controlled temperature, light, or humidity. After 3 days  from transplantation, plants were treated with  *T. harzianum*  (1 × 10⁷ spores mL⁻¹) under the following conditions: (i) non-treated control (NT); (ii) inoculated with a suspension of  *T. harzianum* grown in PDB and resuspended in distilled water (TT); and (iii) inoculated with a suspension of  *T. harzianum*  grown in PDB supplemented with 6% gazpacho (TG). For each treatment, ten plants (replications) were used, with pots arranged randomly to ensure uniform greenhouse conditions for plants. After inoculation treatment and until at the end of the trial, all plants were supplied with tap water, in order to keep the growth substrate moisture at the field capacity (FC) level.

At 31 days after inoculation, five plants per treatment were used for the assessment of leaf number and plant height. Moreover, after biometric measurements, these plants were harvested, carefully separated from the soil, washed to remove residual soil particles, and dried at 60 °C until the constant weight, to determine dry biomass.

Data was analyzed using one-way ANOVA performed with STATSoft software. Results are presented as the mean (n = 5) ± standard deviation (SD). Of the five plants measured, the highest and lowest values were excluded from the calculation of the mean and from statistical analysis, to maintain an equal number of replicates for all groups.

**Table S6.** Biometric measurements from 5 plants per treatment, after 31 days from the application of the *T. harzianum* inoculum. The number of the replicates is indicated as R1, R2, and R3.

| **Treatment** | **Nr leaves** | **Plant height (cm)** | **Dry biomass (g)** |
| --- | --- | --- | --- |
| NT_R1 | 99 | 18.00 | 2.10 |
| NT_R2 | 106 | 22.00 | 5.00 |
| NT_R3 | 90 | 20.00 | 1.00 |
| NT_R4 | nd* | nd | nd |
| NT_R5 | nd | nd | nd |
| **Mean** | **98.33 ± 8.02** | **20.10 ± 2.00 b** | **3.00 ± 2.50** |
| TT_R1 | 116 | 21.70 | 3.20 |
| TT_R2 | 100 | 20.70 | 2.90 |
| TT_R3 | 110 | 19.50 | 3.50 |
| TT_R4 | 97 | 17.00 | 2.10 |
| TT_R5 | 115 | 22.60 | 4.80 |
| **Mean** | **107.6 ± 8.67** | **20.60 ± 1.20 b** | **3.30 ± 1.00** |
| TG_R1 | 92 | 26.50 | 5.00 |
| TG_R2 | 104 | 24.00 | 5.00 |
| TG_R3 | 93 | 25.40 | 5.30 |
| TG_R4 | 70 | 23.00 | 4.40 |
| TG_R5 | 105 | 28.00 | 7.70 |
| **Mean** | **92.80 ± 14.10** | **25.40 ± 2.00 a** | **5.50 ± 1.30** |

*nd: plant was lost during the experiment

**Table S7.** One-way ANOVA test on height, leaves number and dry biomass of tomato seedlings harvested after 31 days from inoculation.

| One-way ANOVA | **Nr leaves_(T31)** | **Plant height (T31)** | **Dry biomass (g)** |
| --- | --- | --- | --- |
| df, *p value* | Df: 2, p> 0.05 | Df: 2, p< 0.05 | Df: 2 p> 0.05 |

**Figure S1**. Scatterplot as a mean (n=5) ± SD of leaves number, height and dry biomass of tomato seedlings after 31 days from inoculation treatment. Different letters indicate statistical differences between culture media, according to Tukey’s test (p ≤ 0.05).


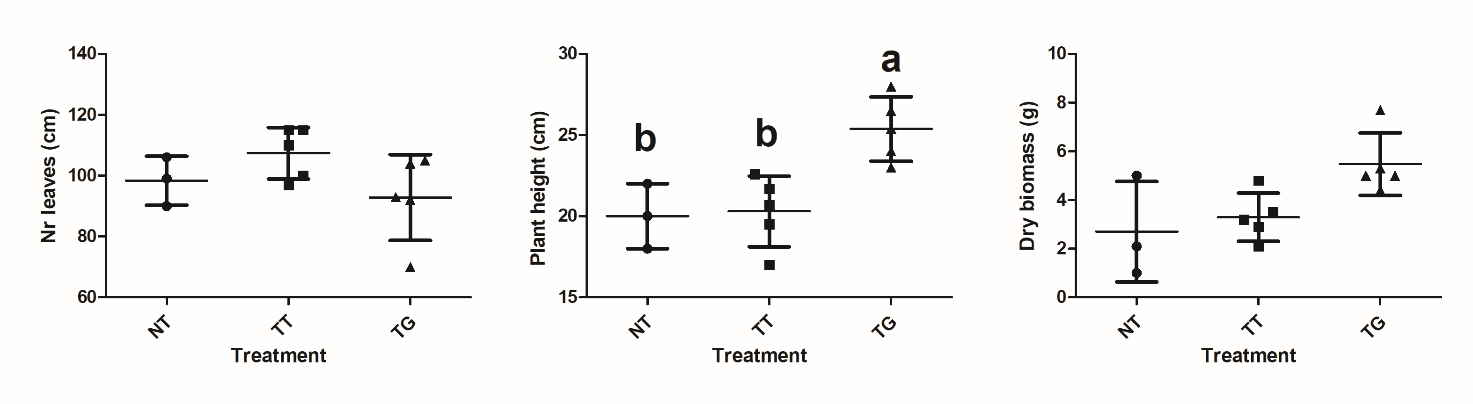


Preliminary conclusions

The data indicates that tomato seedlings inoculated with T. harzianum  grown in PDB supplemented with 6% gazpacho (TG) differed from those inoculated with T. harzianum  grown in PDB alone (TT) in terms of plant height. Specifically, plants treated with the TG inoculum were significantly taller than those treated with the TT inoculum. Although not statistically significant, plant biomass was also higher in TG-inoculated plants compared with TT-inoculated plants, as shown in the table. This lack of significance is likely due to the substantial variability in the biometric data of untreated plants, which reduced the ability to detect differences between treatments. For this reason, the experiment will be repeated next spring. Overall, the differences observed between TG- and TT-inoculated plants are encouraging and support the potential use of Trichoderma grown in PDB supplemented with 6% gazpacho.
