# Supplementary figures and images for: Evaluation of tomato based agro-industrial byproducts as substrates for Trichoderma harzianum cultivation and bioinoculant potential
Source: Front Microbiol. 2026 Jan 16;16:1713960. doi: 10.3389/fmicb.2025.1713960 (PMC12858247; doi:10.3389/fmicb.2025.1713960)

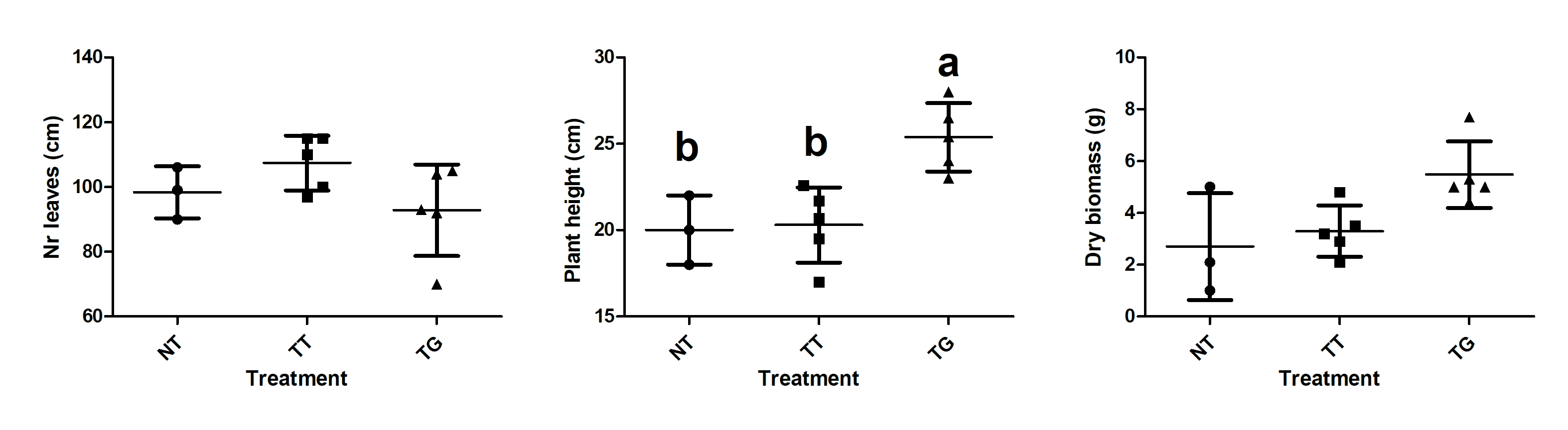

Supplement: Supplementary file 1 [file Data_Sheet_1.ZIP › supplementary materials S1/Figure S1.tif]
